# The role of extracellular vesicles in vascular calcification in chronic kidney disease

**DOI:** 10.3389/fmed.2022.997554

**Published:** 2022-10-28

**Authors:** Huan Zhao, Haojie Liu, Yueming Liu, Juan Jin, Qiang He, Bo Lin

**Affiliations:** ^1^Urology and Nephrology Center, Department of Nephrology, Zhejiang Provincial People’s Hospital, Affiliated People’s Hospital, Hangzhou Medical College, Hangzhou, Zhejian, China; ^2^Department of Nephrology, The First Affiliated Hospital of Zhejiang Chinese Medical University (Zhejiang Provincial Hospital of Traditional Chinese Medicine), Hangzhou, Zhejiang, China

**Keywords:** extracellular vesicles, exosomes, vascular calcification, chronic kidney disease, cardiovascular disease

## Abstract

Widespread vascular calcification (VC) in patients with chronic kidney disease (CKD) is the pathological basis for the development of cardiovascular disease, and VC has been identified as an independent risk factor for increased cardiovascular mortality in cases of CKD. While VC was earlier thought to be a passive deposition process following calcium and phosphorus supersaturation, recent studies have suggested that it is an active, modifiable, biological process similar to bone development. The involvement of extracellular vesicles (EVs) in the process of VC has been reported as an important transporter of material transport and intercellular communication. This paper reviews the mechanism of the role of EVs, especially exosomes, in VC and the regulation of VC by stem cell-derived EVs, and discusses the possible and promising application of related therapeutic targets in the clinical setting.

## Vascular calcification

Vascular calcification (VC) is the process of abnormal deposition of minerals such as calcium and phosphorus in the form of hydroxyapatite within the vessel wall (1). The prevalence of calcification increases with worsening kidney disease (2), by the time patients reach the dialysis stage, 70–80% have significant coronary artery calcification (3). VC occurs in two different phenotypes, medial and intimal calcification, differing in their pathogenesis (4, 5). While intimal calcification is mainly inflammation-driven and closely associated to atherosclerotic plaques, medial calcification is considered to be the major form of VC in CKD (4, 6).

**FIGURE 1:**
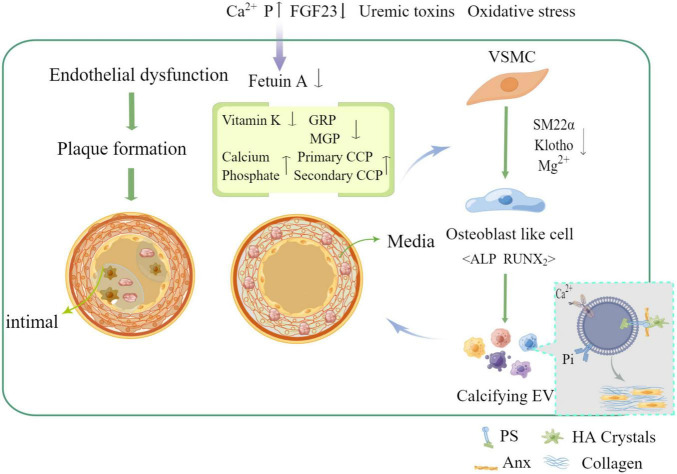
Mechanism of vascular calcification formation and related influencing factors (by Figdraw).

It has been shown that the main cause of medial and intimal calcification is the phenotypic transformation of vascular smooth muscle cell (VSMC), which occurs through many different stimuli such as hyperphosphatemia, hypercalcemia, and disorders of mineral metabolism in the context of uremia, hyperphosphatemia, and overuse of calcium binding agents (7). In contrast to hypertension, dyslipidemia, inflammation, and other compared to traditional risk factors, phenotypic transformation of VSMC has become an important risk factor for VC in chronic kidney disease (CKD) (8). Also, homeostatic imbalance between calcification-inhibiting and inducing factors [e.g., pyrophosphate, Fetuin-A, osteoregulin (OPN), osteoprotegerin (OPG), and vitamin k-dependent matrix vitronectin (MGP) and vitronectin-rich protein (GRP)] leads to extracellular matrix (ECM) mineralization (9) (as shown in Figure 1). Recent studies have suggested that intercellular communication pathways through extracellular vesicles and microRNAs may be a key mechanism in VC, providing a promising area of insight into its pathogenesis (10).

## Extracellular vesicles

### Overview of extracellular vesicles

**FIGURE 2:**
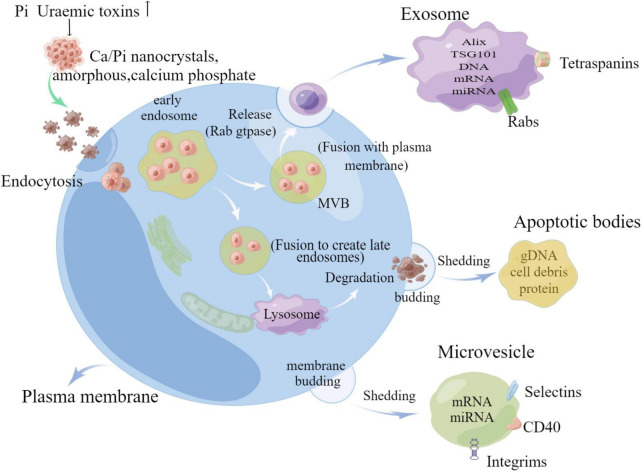
Schematic diagram of extracellular vesicle release (by Figdraw).

Extracellular vesicles (EVs) are ECM derivatives, which are membrane-bound particles released by cells. EVs contain multiple forms of vesicles, which can be classified into four subtypes, exosomes, microvesicles, apoptotic vesicles, and matrix vesicle, based on the biogenesis pathway, size, density, and protein markers of EVs, and the main characteristics of various EVs are shown in Table 1 (11). Not only that, different types of EVs contain different forms of basic substances, including proteins, carbohydrates, and lipids, in addition to various types of genetic material, such as DNA and mRNA, and small RNAs, such as microRNAs (miRNAs) (12). Exosomes are produced by a complex system of endosomes that store various luminal vesicles that are guided to reach the lysosome or cell membrane under different circumstances (11). The endosome system can be further divided into three parts: early endosome, late endosome, and circulating endosome. Early endosome becomes late endosome through a series of transformations. Late endosome includes multiple vesicles, also known as multivesicular bodies (MVBs) (13). It can fuse with either lysosomes or plasma membranes, with the former targeting certain proteins or lipids to degrade endosomal contents, while fusion with plasma membranes releases its enclosed exosomes in a Rab gtpase-dependent manner (14). However, microvesicles are directly budded and fissioned by the plasma membrane, and not only that, apoptotic cells are induced to fuse with the plasma membrane due to the disruption of the cytoskeleton and separate to form apoptotic vesicles (15) (as shown in Figure 2).

**TABLE 1:** Main characteristics of exosomes, microvesicles, apoptotic bodies, and matrix vesicles.

	Exosomes	Microvesicles	Apoptotic bodies	Matrix vesicle
Size	30–150 nm	100–1,000 nm	50–5,000 nm	30–300 nm
Origin	Fusion of late endosomal vesicle with plasma membrane	Plasma membrane	Plasma membrane and endoplasmic reticulum of apoptotic cells	Plasma membrane or intraluminal vesicles within multivesicular bodies
Density	1.13–1.18 g/mL	1.16–1.19 g/mL	1.16–1.28 g/mL	
Markers	CD9, CD63, CD81, Rab, annexin, GTPase, flotillin, ESCRT, ALIX, TSG101, VPS4, Hsp70, Hsp90, etc.	CD40, CD31, CD34, CD83, TSG101, ARF6, Integrin, VCAMP3	Annexin V, TSP, C3b, caspase3, phosphatidylserine	Enriched in annexins, MHC I,LAMP-1, LAMP-2, TSG101

Exosomes play an important role in intercellular signaling by protein and nucleotide delivery, bringing about corresponding phenotypic changes and responses in recipient cells, and also in many diseases (16). EVs have been reported to regulate the interaction between hosts and pathogens, affecting a wide range of diseases, including infectious and inflammatory, neurological, and cancer, and involving multiple mechanisms (17). The methylation-modified RNA-binding protein YBX1 promotes lung cancer proliferation and metastasis by regulating oncogenic factors in EVs-related fragments (18). Similarly, β-catenin in renal tubular cells is caused by exosomes, and genetic deletion of this protein greatly ameliorates renal fibrosis (19). In the mid-1960s, Bonucci reported the involvement of EVs in the calcification process (20). Under physiological conditions, EVs can be released from mast chondrocytes, osteoblasts, dentin-forming cells, and tendon cells. In addition, some non-skeletal tissue cells, such as VSMC, dead macrophages, and valvular interstitial cells also release pathological EVs (21). Different types of cells secrete exosomes into body fluids and various microenvironments through various forms such as paracrine secretion and act in adjacent regions or distant cells (22).

### Regulation of extracellular vesicles in vascular calcification formation

**FIGURE 3:**
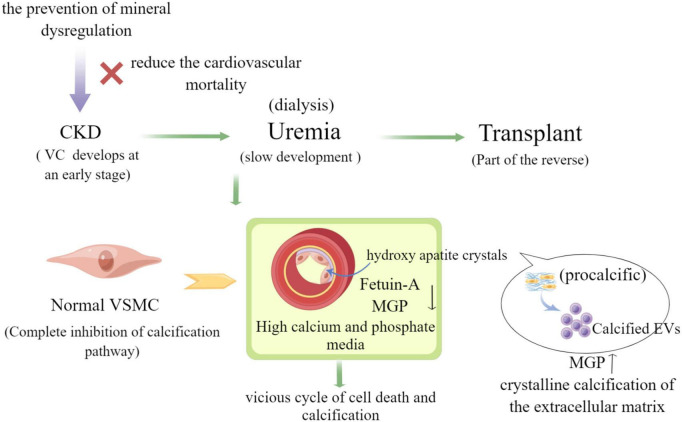
The role of extracellular vesicles in vascular calcification in CKD (by Figdraw).

CKD patients develop extensive and progressive VC that contributes to the high cardiovascular morbidity and mortality. Features such as loss of anticalcific mechanisms, VSMC differentiation, chronic inflammation, increased ECM remodeling, and the release of EVs are known features involved in the development of calcific lesions (10). EVs are a new player in the molecules of intercellular signaling mechanisms, and their role in VC has attracted much attention in recent years from both domestic and international scholars. Some studies have confirmed that EVs derived from smooth muscle cells, stromal cells and macrophages with activity play a key role in heart valve plaque calcification and AS (23), and EVs are also involved in physiopathological processes such as inflammation, proliferation, thrombosis, vasoactive responses, and in the development of AS lesions that promote VC (24). Biological CPPs and EVs were isolated from healthy and CKD patients and comparatively characterized using ultrastructural, analytic, molecular, and immuno-based techniques. VSMC calcification assays reveal that CPPs CKD stage 5 and EVs CKD stage 5 are taken up by VSMC and induce VC by promoting cell osteochondrogenic differentiation and inflammation (7). Furthermore, it has been demonstrated that MVS derived from cells isolated from CKD rat VSMCS can promote calcification of normal rat receptor VSMCS, which is mainly caused by inducing cell signaling changes and phenotypic alteration of recipient VSMC (25). VSMC phenotypic transformation is a key event in the VC process in which exosomes play an important role. Exosomes released from VSMC are the smallest molecules that form microcalcifications and serve as centers for ECM aggregation, which then gradually form mature minerals in CKD (26). In parallel, exosomes have also been found to deliver intracellular contents such as miRNAs and proteins, regulate cellular signaling pathways and processes such as autophagy and oxidative stress responses, and promote VC through signaling (27). Much of our knowledge of the role of EVs in cardiovascular calcification relies heavily on previously established evidence of MVs involved in physiological bone mineralization. Although research in this field dates back to the 1970s, the role of EVs in pathological calcification is still largely unknown (28). Progression of this field has been hindered by the lack of sophisticated modalities to visualize EVs *in vitro* and *in vivo* (as shown in Figure 3).

#### Extracellular vesicles promote vascular calcification by regulating mineral deposition through calcium and phosphorus homeostasis

Mineral dysregulation, especially phosphate accumulation, plays a key role in the course of VC, and it is well known that inorganic phosphate mediates VC in a time- and dose-dependent manner and is frequently elevated in patients with CKD (29). As glomerular filtration rate gradually decreases and renal phosphorus excretion becomes abnormal in CKD patients with progressive disease, phosphorus accumulates in the body and acts as an intracellular secondary messenger, activating several molecular pathways associated with bone formation, entering the cell through the sodium-phosphorus co-transferron (PiT-1) and playing an important negative role. High levels of intracellular phosphate can increase the bone-specific transcription factor Cbfa1, which activates several osteogenic factors, including bone morphogenetic proteins BMPs (e.g., BMP-2), leading to VSMC differentiation toward an osteoblast phenotype (30). High concentrations of phosphate and calcium promote VC by stimulating osteoblast or chondrocyte differentiation, vesicle release, apoptosis, and degradation of ECM. calcified EVs (100—300 nm) released from macrophages and smooth muscle cells act as foci of mineralization nucleation, forming spherical or ellipsoidal microcalcification foci, which merge to form large calcification foci (≥ 50 μm) (31), as shown by electron microscopy. The initial calcifications formed by exosomes are localized close to collagen fibers and elastin and act as scaffolds to guide the shape and size of calcifications during growth, they vary in size and mineral content and appear to originate from apoptotic or live VSMC in mineralized sites, as well as VSMC, macrophages, endothelial cells, and platelets in AS plaques (32). Under physiological conditions, normal VSMC effectively prevent calcification by expressing calcification inhibitors, some of which are loaded into the exosomes, such as endogenously expressed matrix glycoprotein (MGP) and fetuin-a (fetuin-a), which prevent mineral nucleation (23), but fetuin-a levels are significantly reduced in patients with calcification, and chronic mineral imbalance or inflammation leads to a huge depletion of MGPs and fetuin-a in exosomes and enrichment through proteolipid complexes composed of phosphatidylserine (PS) and membrane-linked proteins (Anx), which convert exosomes into primary foci for calcification by providing mineral nucleation sites (33). Annexin A1 can directly promote the aggregation of EV-EV and actively contribute to the nucleation of mineralized foci, in addition, under pro-inflammatory conditions of high organophosphate, membrane associate A1 can convert pyrophosphate to inorganic phosphorus by enhancing tissue non-specific alkaline phosphatase (TNAP) activity, further increasing the concentration of extracellular free inorganic phosphorus and thus promoting the calcification process (31). Sphingomyelin phosphodiesterase 3 (SMPD3) is a key regulator involved in the production of EVs. An *in vitro* study found that the expression of SMPD3 increases when cells are exposed to high phosphorus and calcium conditions, and that inhibition of SMPD3 hinders exosome production and calcification (33). When intracellular Ca2+ levels are too high, exosomes can take up calcium, phosphorus and other mineral complexes to maintain the homeostasis of intracellular mineral metabolism, which leads to their calcification (34).

#### Extracellular vesicles regulate vascular calcification through phenotypic switching of vascular smooth muscle cell

VSMCs provide structural support and contractile function to the vasculature. In normal adult vessels, they have a low proliferation rate and express their unique contractile proteins. When vascular damage is repaired, vascular remodeling occurs under altered hemodynamics, or in disease states, VSMCs dedifferentiate into a synthetic phenotype that secretes exosomes, a process known as phenotype switching (8). Several lesions of the vascular system have been associated with phenotypic conversion of VSMC, such as AS, restenosis, and VC (35). EVs produced by VSMC under physiological conditions do not contain calcium phosphate crystals and also transport calcification inhibitory proteins such as vitamin k-dependent MGP and fetuin-A (28). If this calcium phosphate stimulation phenomenon, which occurs in CKD patients with elevated phosphate levels, calcium phosphate crystals are loaded into EVs (36). Phenotypic conversion of VSMC is a key aspect of VC development. VSMC can be converted from a contractile phenotype to a synthetic phenotype, facilitating the release of EVs and converting them to a calcified state (37). Calcified EVs contain fewer inhibitors of calcification, and when released into the ECM, it tends to aggregate and form microcalcifications in areas of sparse collagen. Sortlin increases the calcification of EVs by regulating the entry of TNAP into them (38). Meanwhile, during the process of transdifferentiation under calcified VSMC-derived EVs, VSMC exhibited decreased expression of the contractile markers smooth muscle α-actin (α-SMA), smooth muscle 22α (SM22α), SM-MHC, CNN1, calcium regulatory protein, smooth muscle protein, SMTN, myosin light chain (MYL) and decreased expression of the synthetic markers S100A4, KLF4, waveform protein, and OPN were increased, which was also associated with increased synthetic cytosolic calcification in response to high extracellular calciumions (39), while runt-related transcription factor 2 (Runx2), zinc finger transcription factor (Osterix), myosin homology frame 2 (MSX2), BMP-2, and alkaline phosphatase (ALP), all of which are osteogenic markers, were highly expressed to cause increased intracellular and extracellular calcium deposition, ultimately leading to cellular and VC. In addition, oxidative stress injury, high phosphorus environment, inflammation, and various biochemical factors such as platelet-derived growth factor (PDGF-BB) and transforming growth factor-β1 (TGF-β1) are involved in the expression of bone formation-related indicators and promote the transformation of VSMC to osteoblasts (40), which may all involve the role of calcifying EVs. Furthermore, EVs in the serum of CKD patients are prone to VC because they carry a higher percentage of calcification-related markers, such as GRP (41). It induces receptor calcification of VSMC possibly through the activation of multiple signaling pathways, such as mitogen-activated protein kinases (MAPK and Erk1/2) and NADPH oxidase (NOX) signaling pathways (25). High glucose-stimulated human umbilical vein endothelial cells (HUVECs) can secrete exosomes that induce calcification and senescence in VSMC (42). Not only that, during CKD, the uremic environment in the serum provides conditions for the release of pro-calcification EVs isoforms, and the released EVs promote osteogenic transdifferentiation of VSMC through enhanced AKT signaling pathway and PiT-1 expression, leading to the development of VC and further deterioration of the disease (43). Similarly, Nox5 is a key regulator of VSMC phenotypic transition, with EVs taking up Ca2+ and inducing reactive oxygen species (ROS) production by VSMC *via* Nox5, providing a potential target for regulating vascular remodeling and calcification in the context of mineral imbalance (39). In summary, EVs are involved in regulating the formation and development of VC, which may be closely related to VSMC osteoblast phenotypic transformation, while the release of EVs generally occurs in the context of VC, so interfering with the release of EVs may also be an effective way to improve VC. In addition, the mechanism of calcified EVs formation also needs to be further explored and investigated.

#### Extracellular vesicles regulate vascular calcification by mediating mi ribonucleic acids as well as driving-related signaling pathways

The VSMC calcification process is tightly regulated by gene reprogramming, and as such, there is growing evidence to support an integral role of miRNAs in this process. miRNAs are small non-coding RNAs that are generally transcribed from DNA into primary miRNAs and then processed into precursor miRNAs and mature miRNAs. They usually have translational regulatory functions. miRNAs inhibit target mRNAs to negatively regulate gene expression mainly by incomplete base pairing with the 3’ untranslated region of target mRNAs, thereby inhibiting protein production or mRNA degradation, and play roles in many biological processes such as development, cell proliferation and differentiation, apoptosis and immune regulation (44), and also play important regulatory roles in pathology such as CKD and related complications, especially VC. EVs contain an environment-dependent load, such as calcification inhibitors and miRNAs, and thus act as carriers or communicators between cells to influence cellular behavior, including VSMC calcification. Studies have shown that miRNAs with elevated expression during VC can promote vascular VSMC osteogenic-like transformation by targeting anti-calcification proteins or contractile markers, while other miRNAs with reduced expression inhibit VSMC osteogenesis by targeting osteogenic transcription factors (45). Among the miRNAs loaded in calcified EVs, a large number of them are associated with osteogenic differentiation, such as miRNAs 30, 125-b, 143, 145, and 155 all affecting the expression of specific osteogenic markers (Smad1, RUNX-2, ALP, and bone sterix), and altered concentrations of these miRNAs in EVs lead to altered calcium and MAPK signaling pathways and are associated with SMCs-mediated calcification (24). miR-221 and miR-222 expression was significantly enhanced in uremic rat EVs, while the corresponding control regulates the post-transcriptional contractile VSMC phenotype miR-143/145 was significantly reduced, and these EVs could be involved in VSMC phenotypic transition and calcification by enhancing AKT signaling and PiT-1 expression (43). miR-155 is critical in the VC process, and exosomal miR-155 from T cells may serve as an additional source of miRs during the VC process, thus suggesting that exosomes may be a new point of interaction between the immune response and the VC process, and are dependent on the transport of miRs (46). Circulating miR-223 is downregulated in CKD patients, and reduced miR-223 expression is thought to be a risk factor for the development of VC by a mechanism involving its possible packaging into exosomes (47). Similarly, EVs from hemodialysis patients express surface proteins and carry miRNAs involved in inflammation-related endothelial dysfunction and VC, significantly reducing the expression of EVs-derived miR-223 (48). The interaction between endothelial cells and SMCs also plays a key role in maintaining vessel wall homeostasis, and exosomes are important carriers of cell–cell interactions and information transfer under normal and pathological conditions. Notch3-loaded exosomes have been reported to communicate between the two cell types and to be involved in the mTOR signaling pathway (49). Circulating EVs, such as those from platelets, monocytes/macrophages, and ECs, were significantly increased in most hemodialysis patients compared with healthy subjects, with only circulating EVs from endothelial cells, which may suggest that circulating EVs in CKD patients are mainly driven by ECs (48). These EVs promote VSMC calcification and cause endothelial dysfunction *in vitro* by a mechanism associated with upregulation of EVs-loaded miR-223 levels. Notably, ECs apoptosis-triggered endothelial particles (EMPs) can reduce VSMC proliferation and migration, and reduce neointima formation with the transfer of vector miR-126 to VSMC (50). In summary, miRNAs packed in EVs may contribute to VC in two ways. First, vesicles packaged with miRNA for paracrine signaling within the plaque may become entrapped and form microcalcifications. Second, in turn, this may prevent the miRNAs within the vesicles from reaching the intended target cell, leading to phenotypic changes that promote further calcification (51). Therefore, insight into the underlying mechanism of selective packing of miRNAs into EVs and selective uptake into the target cell will help increase understanding of the role of miRNA-containing vesicles in physiological intercellular communication as well as unintended disruption of this communication, which may prevent calcification in the vascular system to delay the CKD process.

## Extracellular vesicles are involved in kidney stone formation in the context of chronic kidney disease calcification

In one study, carotid intima-media thickness was shown to be increased in patients with calcium kidney stones, independent of dyslipidemia, compared with controls without stones (52). Considerable evidence supports that patients with kidney stones are at higher risk for VC. EVs are present at the site of VC and in the papillary region of the kidney (53, 54). Stromal vesicles are extracellular membrane-bound vesicles secreted by various cell types and are involved in the progression of cardiovascular calcification. For kidney stones, crystal deposition in the renal papilla is thought to begin with stromal vesicles, and under conditions of mineral imbalance, calcium and phosphate flow through their appropriate channels into EVs, leading to initial mineral accumulation in the form of amorphous calcium phosphate (55), forming hydroxyapatite crystals; At the same time, EVs release crystals through the membrane to expose preformed hydroxyapatite nanocrystals to the extracellular fluid. Once exposed to this fluid, nanocrystals can act as sites or templates to form new crystals through homologous nucleation (56) and possibly to mineralize ECM components. This region grows in size with additional crystals deposited in the collagen frame (57).

## Extracellular vesicles for vascular calcification in chronic kidney disease

EVs secreted by stem cells are important active substances with immunomodulatory, anti-apoptotic, anti-fibrotic, and angiogenic therapeutic effects similar to those of stem cells themselves, and are more suitable for the treatment of complex diseases due to their stable properties and lower risk of severe immune response compared to cells or cytokines. Its role in many diseases has been studied, such as myocardial ischemia-reperfusion (58), hypoxia-induced myocardial injury (59), healing process of diabetic foot ulcer wounds (60), intervertebral disc degeneration (61), and liver failure (62) have been studied for their protective effects. Stem cell-derived EVs have regenerative potential and potential therapeutic value for VC, however, studies on the effect of MSC-derived exosomes on VSMC VC are scarce. Mesenchymal stem cells (MSCs) are pluripotent stem cells present in various organs and can be obtained from a variety of tissues or cells such as bone marrow mesenchyme, adipose tissue, muscle, skin, fallopian tube tissue, umbilical cord blood, menstruation, and even induced pluripotent stem cells. Their powerful paracrine effects make bone marrow MSCs candidates for endogenous regenerative and repair pathways (63). In addition, EVs can be packaged, modified, and loaded with additional substances that regulate biological activity, such as miRNAs, proteins, and lipids. These stem cell-derived EVs can be taken up by other cells and promote paracrine signaling, thus regulating calcification. Bone marrow mesenchymal stem cell (BMSC)-derived exosomes can also attenuate high phosphorus-induced VSMC calcification by modifying miRNAs profiles (39). Not only that, BMSC-Exos reduced high pi-induced expression of Runx2, osteocalcin and BMP2 and inhibited calcium deposition, NONHSAT 084969.2 and NF-κB pathway may play an important role in BMSC-Exos inhibition of VC (64). In a mouse model of CKD, delivery of BMSC-derived exosomes inhibited phosphate-induced aortic calcification and reduced renal fibrosis, and the application of exosomes resulted in reduced urea levels of BUN, Cre, and FGF23 and improved renal function in the CKD model. Also, the phase 2/3 clinical study of stem cell-derived exosomes in CKD showed improvement in eGFR, creatinine, and inflammation. Not only that, BMSC-derived exosomes affect the expression of several miRNAs, including those involved in Wnt, mTOR, and MAPK signaling pathways, which are associated with VC (65). BMSC-derived exosomal miR-381- 3p can also prevent lipogenic differentiation by downregulating NFAT5, attenuating apoptosis and VC, and a variety of miRNAs are involved in this biological process (66). BMSC-derived exosomal miR-381-3p down-regulates NFAT5 and thereby attenuates apoptosis and VC, and these results suggest that exosomes may regulate VC by affecting phenotypic transformation through transporting miRs (67). Exosomes of melatonin mt-treated VSMC can attenuate VC and senescence in a paracrine manner *via* miR-204/miR-211, a potential target of both miRs may be BMP2 (68). Therefore, understanding the complex regulatory network of exosomal miR-204/miR211 and BMP2 may help develop effective therapies for the prevention of VC and aging.

MVs are endogenous nanovesicles secreted by living cells. they are more suitable as therapeutic drug carriers due to their advantages of small particle size, low toxicity, non-immunogenicity, good permeability, and high targeting. Research has shown that VSMCs-derived MVs have shown positive results in many disease models, such as reducing the size of myocardial infarction and improving the inflammation caused after myocardial infarction, resolving pulmonary hypertension, ameliorating renal fibrosis, and restoring neurovascular functions and plasticity (69). In addition to using MVs as delivery vehicles, the contents of calcifying EVs themselves have also been proven to be an essential source of drug targets (69). Therefore, MVs may be a new treatment for VC. This may be because MVs can induce receptor-mediated signal transduction by surface-binding ligand in the pathological environment. Specific functional proteins and miRNAs are delivered to target cells through cell surface receptors. VC is a cell-mediated process that requires genetic alterations which may at least in part be modulated through miRNA (70). MiRNAs play a prominent role in post-transcriptional gene regulation and can be transferred from cell to cell by EVs (71). MiRNAs have been investigated as therapeutic targets to reduce CKD-associated atherosclerosis. Thus, in mice with renal injury, increased levels of miR-92a-3p were observed and inhibition of miR-92a-3p with a single injection miRNA inhibitors complexed to HDL significantly reduced atherosclerotic lesions (72). Inhibition of these miRNAs significantly altered the TGFβ pathway and STAT3 trancriptional activity (72). Therefore, replacement of protective miRNAs may be a potential therapeutic strategy for VC. One study confirmed that differential miRs under EVs load affect calcification by applying specific miRs inhibitors and miRs mimics (43). These circulating miRNAs may not have biological functions while circulating. Multiple miRNAs can target the same gene (e.g., Runx2–miRNA cluster), and one miRNA might have several targets. Only a small amount of these fine-tuned targets may alter biological responses and phenotypes. Instead, they may act as intercellular communicators, and this communication might be disturbed in calcification disorders, where calcifying matrix vesicles fail to properly deliver miRNAs to the target cell. Studies to fully exploit this potential novel mechanism of calcification are needed (51). Additionally, researchers have successfully used exosomes to carry small molecules, nucleic acids, and proteins to treat various diseases in recent years. It may be possible to use exogenous engineered EVs as a viable therapeutic strategy for VC, whereby inhibitors of VC could be efficiently conveyed into atherosclerotic plaques or vascular microcalcification areas. Although the EVs-based nano drug delivery system showed many significant advantages such as better biocompatibility, homing effect, and convenient for surface modification, it was still in the initial stage for the treatment of disease. There are still many questions and challenges for researchers (73). In order to find out specific treatment methods for VC based on EVs in future studies, we can focus on miRNAs in EVs, use EVs as carriers to develop safer and more effective nanomedicines, and develop miRNA markers to monitor the presence and degree of calcification, so as to better prevent and treat calcification in CKD. In addition, autophagy is involved in the amorphous ca2 + and recycling of Pi or hydroxyapatite, adjust the ca2 + homeostasis of VSMC, will affect the ability of the contraction of the aorta, protect cardiovascular tissues from calcification, however, EV may be in the process of VC, or in the form or release phase, intertwined with autophagy vesicle network, Targeting the molecular mechanisms of autophagy is an attractive therapeutic strategy to inhibit the progression of VC or induce its resolution (15).

## Conclusion

EVs play an important role in both the initiation and progression of VC in CKD. In this paper, we review the regulatory role of EVs in VC, which regulates mineral deposition through calcium-phosphorus homeostasis, induces phenotypic transformation of VSMC, mediates miRNAs, and drives related signaling pathways, and the therapeutic role of EVs in VC; it is worth noting that pharmacological regulation of autophagy and the development of nanomaterials using EVs as vectors may become an exciting new strategy for the treatment of VC. Although many new insights into the pathogenesis of VC have emerged in recent years, many details remain unclear, and therefore, better therapeutic options for patients are limited, and more basic research is needed to translate promising experimental results into routine clinical practice with the aim of developing more new therapeutic targets.

## Author contributions

HZ and HL wrote the manuscript. YL and JJ revised the manuscript. QH and BL selected topic. All authors listed have made a substantial, direct, and intellectual contribution to the work, and approved it for publication.
